# Syndrome of inappropriate antidiuretic hormone secretion and Leser–Trélat syndrome as uncommon paraneoplastic manifestations of renal malignancy – a geriatric experience: a case report

**DOI:** 10.1186/s13256-019-2122-8

**Published:** 2019-06-21

**Authors:** Larry Nyanti, Affizal Samsudin, Ing Khieng Tiong

**Affiliations:** 1 Geriatric Unit, Sarawak Heart Center (Pusat Jantung Sarawak), 94300 Kota Samarahan, Sarawak Malaysia; 2 University Malaysia Sarawak, Kota Samarahan, Sarawak Malaysia

**Keywords:** Leser–Trélat, SIADH, Malignancy, Hyponatremia, Geriatric

## Abstract

**Background:**

Leser–Trélat syndrome, which manifests as eruptive multiple seborrheic keratoses, is a rare paraneoplastic sign. Hyponatremia in the elderly population is an often overlooked but potentially sinister biochemical abnormality. Cancer-related causes of hyponatremia include syndrome of inappropriate antidiuretic hormone secretion, cerebral or renal salt wasting, and adrenal dysfunction. We report a case of an elderly man who presented with both syndrome of inappropriate antidiuretic hormone secretion and Leser–Trélat syndrome, and was eventually found to have renal malignancy.

**Case presentation:**

A 74-year-old indigenous Malaysian man with underlying chronic kidney disease presented with recurrent admissions for hyponatremia with parameters indicative of syndrome of inappropriate antidiuretic hormone secretion, constitutional symptoms, and diffuse skin lesions suggestive of multiple seborrheic keratoses. A radiological workup revealed metastatic renal cell carcinoma with evidence of metastasis to the brain, adrenal glands, bone, and lungs.

**Conclusions:**

To the best of our knowledge, renal malignancy presenting as syndrome of inappropriate antidiuretic hormone secretion and Leser–Trélat concurrently is rare. The causes of hyponatremia in the elderly, approach to investigation, and value as a poor prognostic marker in malignancy are highlighted. We also discuss Leser–Trélat syndrome, its pathophysiology, and its possible implications on clinical practice.

## Background/Introduction

Hyponatremia is a common occurrence among the elderly, but should not be overlooked as it is a poor prognostic marker in malignancy. Leser–Trélat syndrome is a dermatological paraneoplastic sign rarely associated with renal malignancy [1]. We report a case of a 74-year-old man who presented with recurrent admissions for hyponatremia, complaining of constitutional symptoms and diffuse skin lesions suggestive of multiple seborrheic keratoses. A subsequent workup revealed metastatic renal cell carcinoma with evidence of metastasis to the brain, adrenal glands, bone, and lungs. We discuss the possible causes of hyponatremia in the elderly, its association with malignancy, and approach to investigation. While Leser–Trélat syndrome is rare, its pathophysiology and implications on clinical practice are also highlighted. Clinicians are alerted to the importance of thorough investigation of hyponatremia in the elderly population.

## Case presentation

A 74-year-old indigenous Malaysian man, an ex-smoker of tobacco, with underlying severe aortic stenosis, atrial fibrillation, hypertension, and chronic kidney disease presented to us with a 6-month history of lethargy, subjective loss of weight, loss of appetite, and night sweats associated with a sharp, persistent right-sided headache and left hip pain. He denied any pruritus. He denied chronic cough, and had no significant travel history or high risk behavior. He had no family history of malignancy. He had no baseline ultrasound of the genitourinary tract, having refused investigation of his chronic kidney disease previously.

Prior to this current admission, he had been admitted two times over the past 4 months for hyponatremia and normochromic normocytic anemia, with initial serum sodium measuring 120 mmol/L and 124 mmol/L, respectively. Peripheral blood film showed features suggestive of iron deficiency anemia; concurrent with a serum iron level of 9.7 umol/L with calculated transferrin saturation of 21.2%. Ferritin and B12 levels were normal while a fecal occult blood test was negative. On both occasions, he received intravenously administered saline and subsequently was discharged with orally administered sodium supplements. Tests for thyroid function and cortisol levels from a previous admission were normal.

On admission, he was afebrile with a blood pressure of 130/70 and pulse rate of 70. An examination revealed a mildly cachexic man with generalized disuse atrophy of all limbs. Functionally, he was unable to ambulate due to left hip pain. There was tenderness at his left hip with reduced passive and active movement due to pain, and a bony protuberance at the posterolateral aspect of the right side of his scalp. There were no neurological deficits, and respiratory and abdominal examinations were unremarkable.

**Fig. 1:**
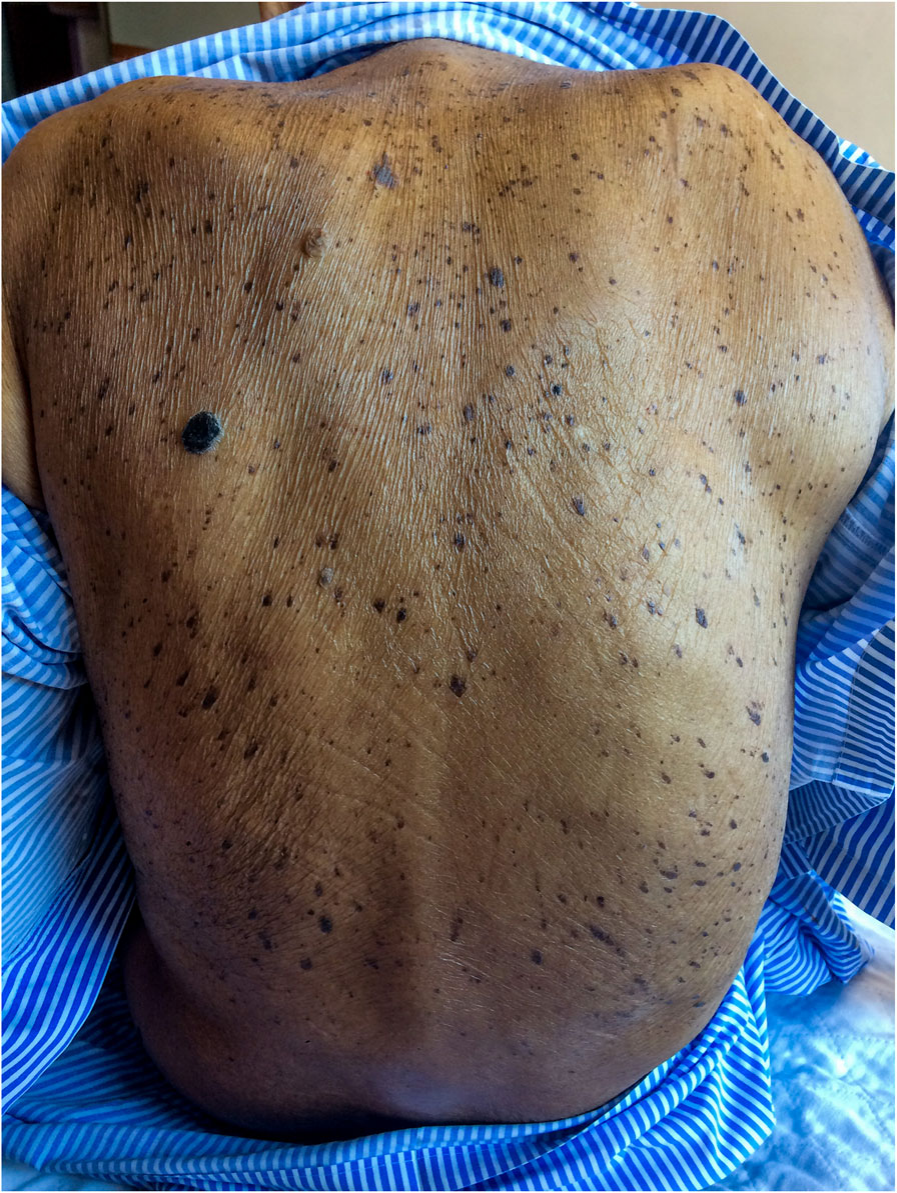
Multiple seborrheic keratosis over the posterior trunk

On examination of the skin, there were diffuse brown well-circumscribed pigmented lesions of undetermined onset over his face and trunk suggestive of multiple seborrheic keratoses. The lesions over his posterior trunk were distributed in a symmetrical “Christmas-tree” pattern (Fig. 1).

**Fig. 2:**
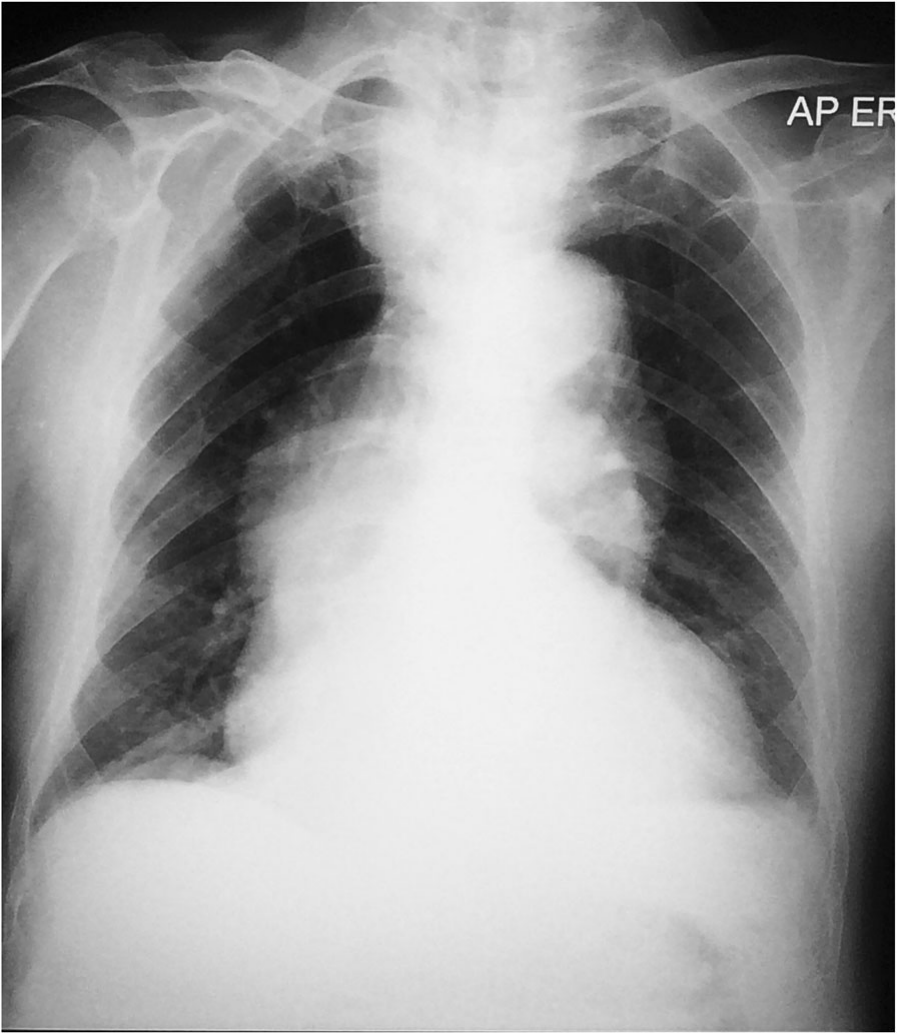
Chest radiograph (anteroposterior view) shows prominent hilar opacities

**Fig. 3:**
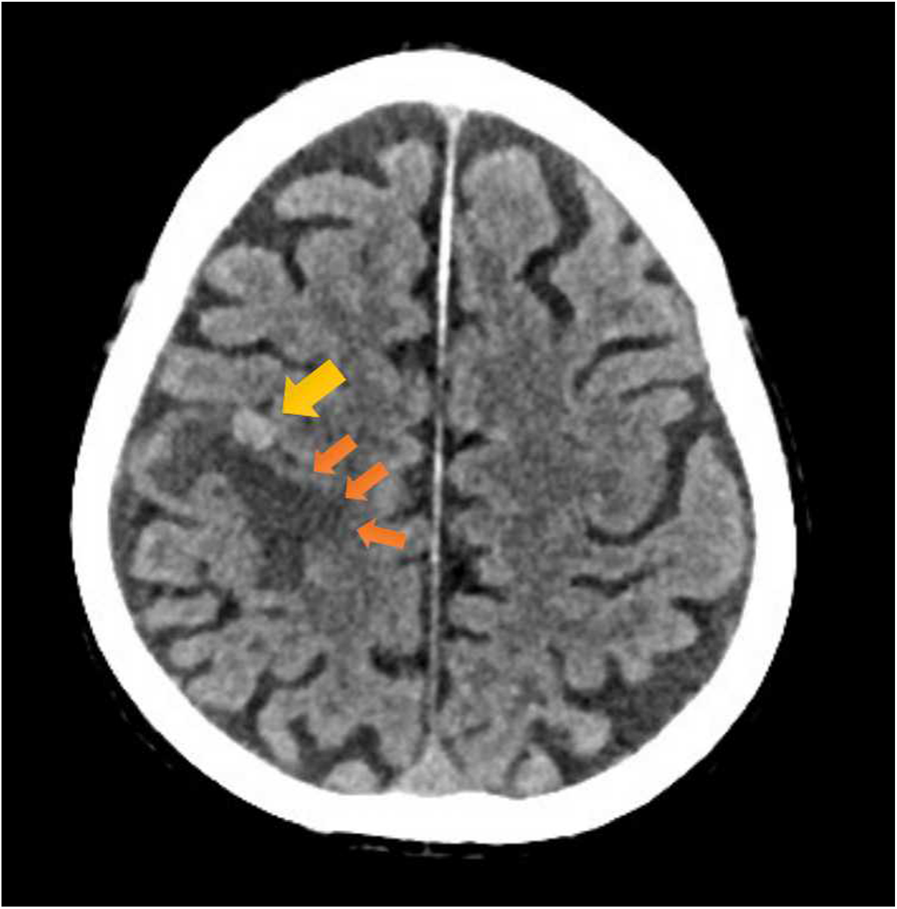
Axial view of non-contrasted computed tomography scan of the brain shows a hyperdense right frontal metastatic brain lesion (*yellow arrow*) with associated perilesional edema (*orange arrows*)

**Fig. 4:**
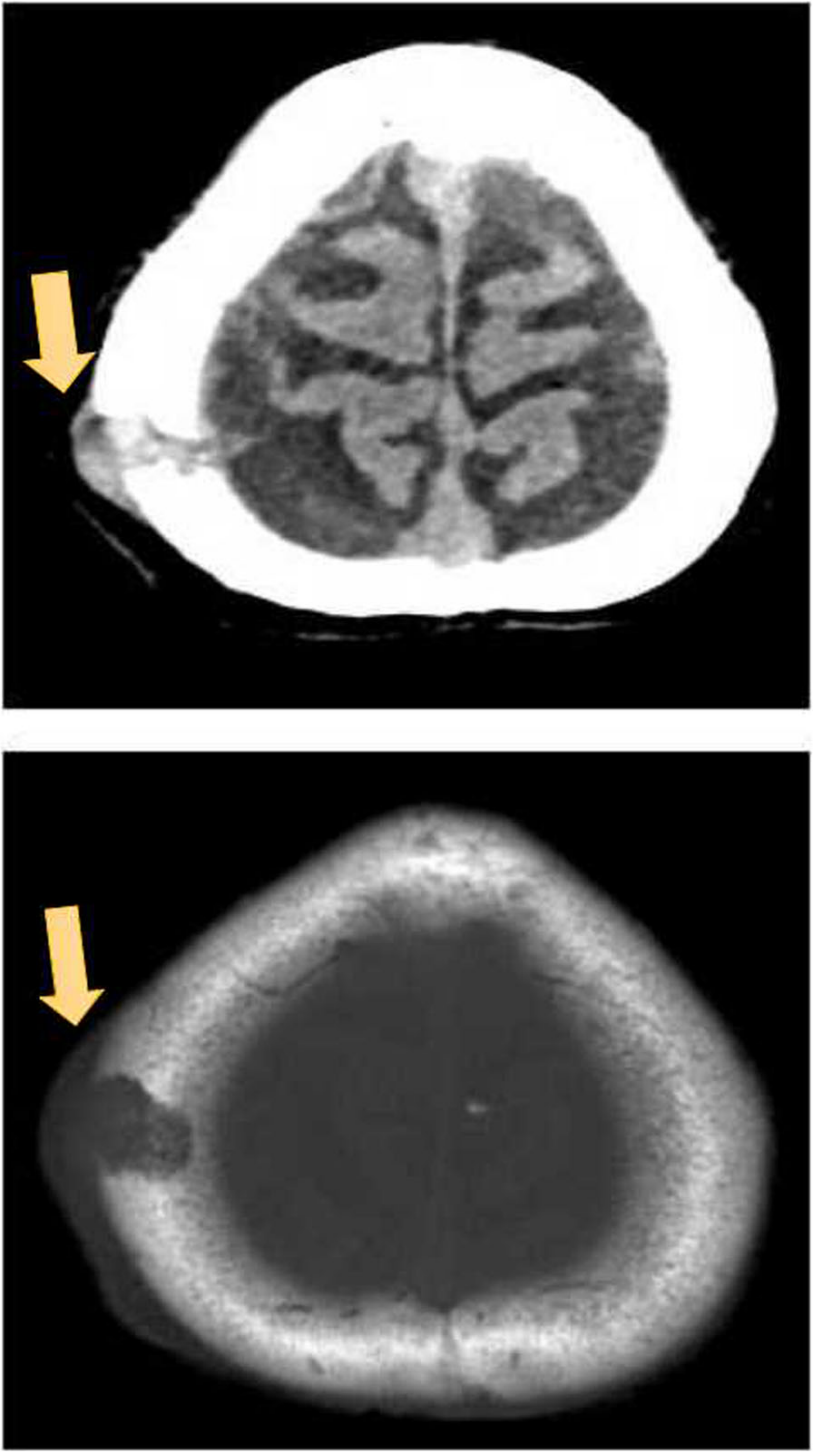
Axial view of non-contrasted computed tomography scan of the brain shows a right parietal bone lytic lesion with soft tissue component (*orange arrows*)

**Fig. 5:**
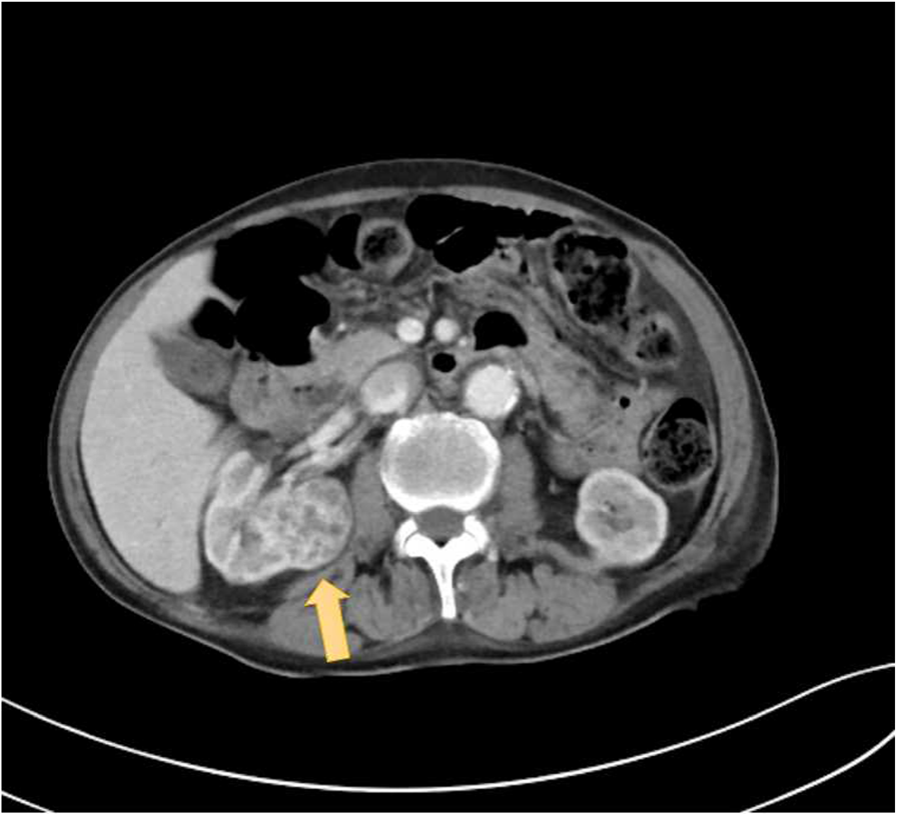
Axial view of contrasted computed tomography scan of the abdomen shows a heterogeneously enhancing right renal mass (*orange arrow*)

**Fig. 6:**
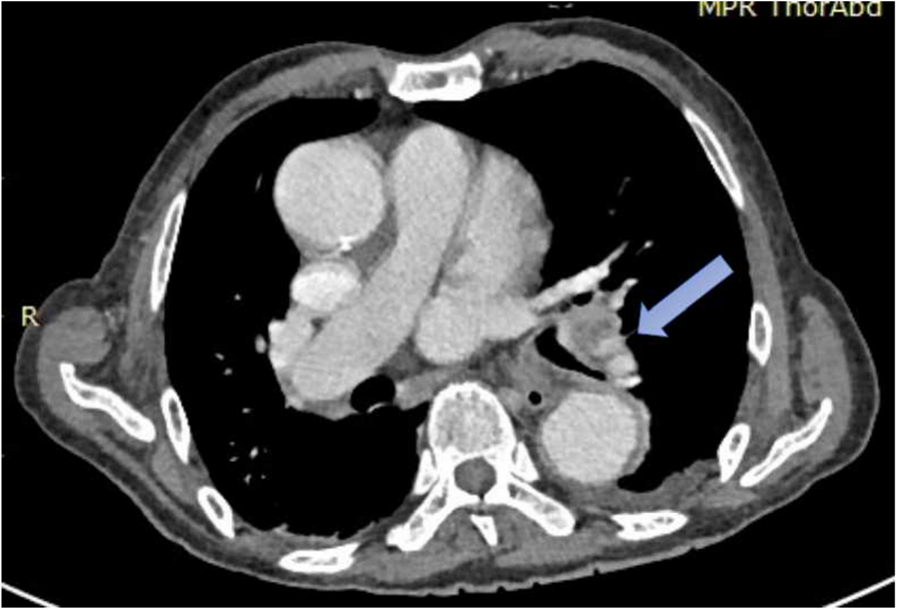
Axial view of contrasted computed tomography scan of the thorax shows an enlarged left hilar lymph node (*blue arrow*)

**Fig. 7:**
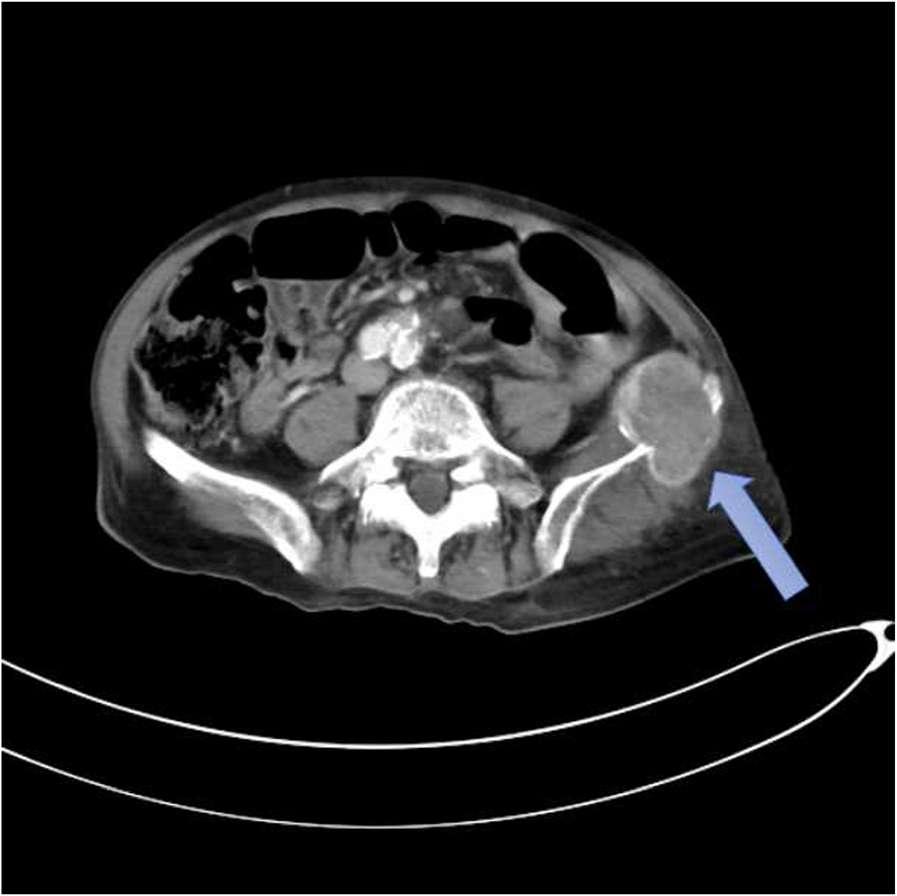
Axial view of contrasted computed tomography scan of the pelvis shows a lytic lesion at the left ilium (*blue arrow*)

An electrolyte investigation showed low serum sodium of 119 mmol/L. All other electrolytes were within normal range, and there was no derangement of liver enzymes. A chest X-ray on admission showed bilateral multiple irregular nodules and prominent hilar opacities (Fig. 2). In view of long-standing headache, we proceeded with non-contrasted computed tomography of his brain, which revealed a well-defined round hyperdense lesion at the right high frontal cortex measuring 0.7 × 0.7 cm, associated with perilesional edema (Fig. 3). There was a lytic lesion at the right parietal bone with expansile soft tissue component (Fig. 4). Resource limitations precluded a follow-on magnetic resonance imaging (MRI). Computed tomography of his thorax, abdomen, and pelvis revealed a heterogeneously enhancing soft tissue mass at the right kidney measuring 3.8 × 4.4 × 3.7 cm, with no evidence of obstruction or hydronephrosis (Fig. 5). There was evidence of bilateral metastatic lung nodules, and multiple hilar and paratracheal nodes (Fig. 6). There was no evidence of aortic aneurysm. There was also a 5 × 5cm lytic lesion at the left ilium, consistent with his left hip pain (Fig. 7).

Further biochemical investigation showed increased urine osmolarity of 303 mOsmol/kg and urine sodium of 48 mmol/L; in view of prior normal thyroid function, cortisol level, and clinical euvolemia, we diagnosed him as having syndrome of inappropriate antidiuretic hormone secretion (SIADH). Fluid restriction was commenced and his sodium levels recovered to 131 mmol/L over 5 days. He was referred to the Dermatology team; in view of the findings of metastatic renal carcinoma, the skin lesions were attributed to Leser–Trélat syndrome.

Having been thoroughly counselled on his condition, he refused renal and skin biopsy and was not keen for further intervention. Despite the absence of a histopathological diagnosis, the constellation of clinical and radiological features was suggestive of a renal malignancy with metastasis, and he was referred to the Palliative Care team for further management.

## Discussion

Hyponatremia can be fairly common among the elderly due to comorbidities such as renal, liver, and cardiac dysfunction or endocrinopathies, as a side-effect of medications or a consequence of polypharmacy and poor nutrition [2]. A retrospective study of 608 community-based elderly individuals found that hyponatremia independently contributed to falls, fractures, and hospitalization [3]. Urine sodium, serum and urine osmolarity, thyroid function tests, and serum cortisol measurements are all essential tests in the workup of hyponatremia [4]. Laboratory investigations should not preclude taking a detailed history and carrying out a thorough physical examination, as these remain important cornerstones in eliciting the etiology of hyponatremia.

Malignancy-associated hyponatremia may result as a consequence of poor diet, adrenal dysfunction, renal or cerebral salt wasting, and SIADH secretion, via ectopic arginine vasopressin (AVP) production or chemotherapy-induced stimulation of AVP [4, 5]. Diagnostic criteria of SIADH include euvolemia, high urine osmolarity (> 100 mOsmol/kg) and urine sodium (> 20 mmol/L) with low serum osmolarity (< 275), having excluded hypothyroid and hypoadrenal states [6]. The treatment of SIADH is by fluid restriction, failing which pharmacological therapies such as urea tablets, demeclocycline, lithium, and loop diuretics have been considered [5]. Tolvaptan, a vasopressin receptor antagonist, is a novel agent in treating SIADH; however, its use in the clinical setting remains limited by its high costs and potential risk of overcorrection [6]. While a clear association of SIADH has been made with small cell lung cancer, the link with renal carcinoma remains unclear, with some authors postulating renal sodium exchange dysfunction as a possible mechanism which needs to be explored further [7].

Hyponatremia in the setting of malignancy has been found to be a poor prognostic factor in hepatocellular carcinoma, gastric cancer, small cell lung cancer, and, as in our case, renal cell carcinoma [3]. A prospective study of 120 patients found that hyponatremia was an independent prognostic factor for poor performance status, weight loss, survival, and lack of response to treatment [7]. A multicenter retrospective study of 1661 patients with metastatic renal cell carcinoma found that those with hyponatremia had poorer prognostic outcomes in terms of survival and treatment failure compared to those with normal serum sodium [8].

The sign of Leser–Trélat is often reported as an eruption of multiple seborrheic keratoses, often pruritic, associated with occult malignancy such as gastrointestinal, breast, lung, liver, pancreas, and prostate. The name is attributed to Edmund Leser and Ulysse Trélat, two European surgeons who initially attributed gastrointestinal adenocarcinomas with these skin lesions in 1890 [9]. Renal cancer is rarely reported in association with Leser–Trélat. The pathophysiology of this dermatological condition has been attributed to endogenous mediators of hyperproliferative skin disease such as Estimated Glomerular Filtration Rate (EGFR) produced by malignancy leading to development of seborrheic keratosis [10].

It is a dermatological finding that remains a topic of contention; a majority of cases are linked with malignancy but a few had no signs of malignancy despite extensive investigation and follow-up [11]. In an attempt to differentiate these two groups, Heaphy *et al*. [11] suggested a differentiation between “Leser–Trélat syndrome,” defined as a strictly paraneoplastic syndrome, versus the classical “sign of Leser–Trélat,” which may or may not have malignancy despite having the dermatological sign. Other reported associations include pregnancy [12], HIV [13], heart transplant [14], acromegaly [15], and erythroderma [16].

## Conclusions

To the best of our knowledge, renal malignancy presenting as SIADH and Leser–Trélat concurrently is rare. There are a few clinical lessons from this case. First, elderly patients presenting with hyponatremia should always warrant purposeful investigations to elicit the cause. Furthermore, recurrent hyponatremia in the presence of constitutional symptoms should raise clinical alarms of malignancy especially if refractory to hydration and orally administered sodium supplementation. The dermatological sign of Leser–Trélat or Leser–Trélat syndrome may be a warning sign of occult malignancy, and although it remains to be further proven with concrete evidence, it is useful as a clinical bedside tool.

## Data Availability

Not applicable.
